# S64. EXECUTIVE FUNCTION OF CHRONIC SCHIZOPHRENIA PATIENTS IN A SEVEN-YEAR FOLLOW-UP

**DOI:** 10.1093/schbul/sby018.851

**Published:** 2018-04-01

**Authors:** Thalita Fernandes, Thais Martins, Gustavo Mustafé, Diego Mendes, Luiz Fernando Pegoraro, Clarissa De Rosalmei Dantas

**Affiliations:** University of Campinas

## Abstract

**Background:**

Cognitive deficits in schizophrenia are generalized, but memory and executive dysfunction represent the more robust impairments and are strongly associated with adverse social and occupational outcomes. Those deficits are present in all phases of the disease, but their course, particularly in the chronic phase, is less clear. The aim of this study is to investigate changes in the performance of chronic schizophrenia patients in tests of executive function over a seven-year test-retest period.

**Methods:**

We will contact 85 patients with schizophrenia, considered clinically stable in previous year, who participated in a study about the deficit syndrome of schizophrenia in 2009–2010. Back then, they were recruited in two sites: an outpatient service of a general hospital (49 patients) and a community-based mental health service for patients with severe mental illness (36 patients), both in Campinas, Brazil. Patients will be reassessed with the same instruments adopted in the first study: SAPS; SANS; Calgary Depression Scale (CDS); Quality of Life Scale (QLS) and a battery of tests comprising Verbal Fluency Tasks; Digit Span Forward (DSF) and Backward (DSB) and Trail Making Tests (TMT) A and B. Additionally, we included three instruments: PSP, for social functioning; Wisconsin Card Sorting Test (WCST) and London Tower Test (LTT), for executive functions. The Wilcoxon test was used to compare executive performance at baseline and at follow-up. Linear regression was used to test associations between variables. We started the recruitment by the patients originally treated in the outpatient clinic.

**Results:**

We present in this poster partial results. Among the 20 patients re-interviewed the mean age at baseline was 36.9 ± 8.9 years, mean duration of mental illness was 16 ± 10.1 years, 75% were men. They had in mean, 10.7 ± 3.3 years of education, only 20% had any work activity, and 15% were married. Mean length of test-retest interval was 6.9 years (minimum 6 and maximum 7.7). At follow-up, 4 patients had improved their education, but only 3 (15%) had any work activity. Up to now 19 patients completed the cognitive reassessment. We performed a principal components factor analysis (PCA) including DSB, TMT-B and VFT for both baseline and follow-up assessments. PCA yielded a single factor for the set of tests in both assessments, which we named Executive Factor, accounting for 57% of variance in baseline and 51.38% in the follow-up assessment. Factor scores were calculated and then compared: 7 patients had higher scores on Executive Factor in the follow-up and 10 had worse scores but differences were not significant. In the linear regression analysis, we did not find significant associations between performance in executive functions in the follow-up assessment and clinical and psychopathological variables neither at the baseline nor at the follow-up assessment. In the Wisconsin test, approximately 60% of the patients managed to form only up to 01 category, which is considered a bad performance. The mean score in LTT was 57.3 ± 10.6 for movements and 170.4 ± 125.2 for time.

**Discussion:**

The results presented are partial, obtained with a provisional small sample size but they show some interesting trends. In general, there was a group tendency for a slightly worse performance after 7 years of the baseline assessment, but we could identify two groups of patients who differ from that general tendency: one with marked deterioration and one with improvement of executive performance over time. If those initial findings are to be confirmed, our next step will be to investigate characteristics associated to improvement or deterioration of executive performance. That sort of information is of great relevance in the pursuit of recovery for schizophrenia patients.

